# Lipid-specific IgMs induce antiviral responses in the CNS: implications for progressive multifocal leukoencephalopathy in multiple sclerosis

**DOI:** 10.1186/s40478-020-01011-7

**Published:** 2020-08-13

**Authors:** Lorna Hayden, Tiia Semenoff, Verena Schultz, Simon F. Merz, Katie J. Chapple, Moses Rodriguez, Arthur E. Warrington, Xiaohong Shi, Clive S. McKimmie, Julia M. Edgar, Katja Thümmler, Chris Linington, Marieke Pingen

**Affiliations:** 1 Institute of Infection, Immunity and Inflammation, University of Glasgow, Glasgow, G12 8TA UK; 2 Departments of Neurology and Neurosurgery, Mayo Clinic, Rochester, MN USA; 3 Virus Host Interaction Team, Leeds Institute of Medical Research, School of Medicine, Faculty of Medicine and Health, University of Leeds, Leeds, LS9 7TF UK

**Keywords:** Type-I interferon, Interferon stimulated genes, Viral encephalitis, John Cunningham polyomavirus (JCV), Microglia, IgM

## Abstract

Progressive multi-focal leukoencephalopathy (PML) is a potentially fatal encephalitis caused by JC polyomavirus (JCV). PML principally affects people with a compromised immune system, such as patients with multiple sclerosis (MS) receiving treatment with natalizumab. However, intrathecal synthesis of lipid-reactive IgM in MS patients is associated with a markedly lower incidence of natalizumab-associated PML compared to those without this antibody repertoire. Here we demonstrate that a subset of lipid-reactive human and murine IgMs induce a functional anti-viral response that inhibits replication of encephalitic Alpha and Orthobunyaviruses in multi-cellular central nervous system cultures. These lipid-specific IgMs trigger microglia to produce IFN-β in a cGAS-STING-dependent manner, which induces an IFN-α/β-receptor 1-dependent antiviral response in glia and neurons. These data identify lipid-reactive IgM as a mediator of anti-viral activity in the nervous system and provide a rational explanation why intrathecal synthesis of lipid-reactive IgM correlates with a reduced incidence of iatrogenic PML in MS.

## Introduction

Neurotropic viral infections pose a major challenge when considering immune modulatory treatment for patients with autoimmune diseases. One of the most devastating of these consequences is progressive multi-focal leukoencephalopathy (PML), a rare demyelinating disease caused by opportunistic infection of the central nervous system (CNS) by JC polyomavirus (JCV; human polyomavirus 2) [1, 2]. The seroprevalence of JCV in healthy adults is 40–80%, but the vast majority of infections are asymptomatic due to a robust immune response [3, 4].

However, immune-suppressed individuals are at an increased risk of developing PML [5]. This has important implications for the clinical management of primary and secondary immune deficiencies, as well as autoimmune diseases in which immunosuppression is a primary treatment option [4]. The latter include multiple sclerosis (MS), a chronic inflammatory demyelinating disease of the CNS [6], in which PML can develop in association with immunomodulatory disease modifying therapies including natalizumab [7] and fumaric acid esters [8].

Natalizumab is a humanised anti-alpha 4 integrin-specific antibody that significantly reduces disease activity by inhibiting migration of immune cells across the blood-brain barrier (BBB) [9]. However, its use is limited by the risk of patients developing natalizumab-associated PML, which in JCV-seropositive patients exceeds 10 per 1000 after a 6 year treatment period [7, 10]. There are no effective treatments for natalizumab-associated PML, other than plasma exchange to remove natalizumab from the circulation. However, as this itself may trigger immune reconstitution inflammatory syndrome (IRIS) mortality remains high and most survivors are left with severe neurological deficits [10].

Current efforts to counter the threat posed by natalizumab-associated PML focus on risk stratification [10]. This is based primarily on JCV serology, but a report identifying intrathecal synthesis of lipid-specific IgM antibodies as a factor associated with a lower probability of developing natalizumab-associated PML suggests an additional strategy [11]. Here, JCV seropositive patients lacking intrathecal IgM were 60-fold more likely to develop PML than those positive for lipid-specific IgM. Importantly, patients positive for both JCV and intrathecal IgM had similar risk of developing PML as patients seronegative for JCV, suggesting these antibodies substantially contribute to JCV defences in the CNS [11]. Inspired by reports that disease-associated autoantibodies induce expression of type-I interferons (IFNs) in systemic lupus erythematosus [12], we speculated lipid-reactive IgM might act to enhance antiviral activity within the CNS.

We now demonstrate that a subset of human and murine IgM autoantibodies that recognise lipids in the CNS induce interferon-β (IFN-β) in microglia. This response is STING-dependent and mediates IFN-α/β-receptor 1 (IFNAR1)-dependent antiviral activity in all major CNS cell types (neurons, astrocytes, oligodendrocytes and microglia), as demonstrated by inhibition of replication of two unrelated encephalitogenic viruses (Bunyamwera virus (BUNV) and Semliki Forest virus (SVF)) in myelinating cell cultures. Our data provide a logical explanation for the observation intrathecal synthesis of lipid-specific IgM is associated with a reduced incidence of natalizumab-associated PML in MS.

## Materials and methods

### Myelinated spinal cord cultures from rat

Neurospheres were generated from the striata of post-natal day 1 Sprague-Dawley rats. Striata were mechanically dissociated in Leibovitz’s L-15 Medium (Gibco) by glass Pasteur pipette and cultured in neurosphere media [Dulbecco’s modified Eagle medium (DMEM)/F12 (Gibco), supplemented with 0.105% NaHCO_3_, 2 mM glutamine, 1% penicillin-streptomycin (Pen/Strep), 5.0 mM HEPES, 0.0001% bovine serum albumin (BSA), 25 μg/ml insulin, 100 μg/ml apotransferrin, 60 μM putrescine, 20 nM progesterone, and 30 nM sodium selenite (all from Sigma)]. Cultures were supplemented with 20 ng/ml recombinant murine epidermal growth factor (Peprotech) and maintained at 37 °C in a humidified atmosphere of 7% CO_2_. Cells were fed every 3–4 days by addition of neurosphere medium and EGF. After 7 days, or when cells had formed large round neurospheres, the neurosphere suspension was centrifuged at 86 rcf for 5 mins and resuspended in astrocyte media [DMEM (1 g/ml glucose, Gibco) supplemented with 10% foetal bovine serum (FBS) and 2 mM L-glutamine (both Sigma)] before being plated onto poly-L-lysine (13 μg/ml) coated cover slips (13-mm diameter, VWR International). Every 3–4 days, half of the old media was replaced with fresh astrocyte media until cells formed a confluent monolayer.

Sprague-Dawley rats were time-mated. At embryonal development day (E)15.5, spinal cords were extracted from embryos, meninges removed and placed in 1 ml Hank’s Balanced Salt Solution (Sigma). Up to 6 cords/ml were then enzymatically digested with 2.5% trypsin (100 μl/ml) and 1.33% collagenase (100 μl/ml) (both Sigma) for 15 mins at 37 °C. Enzymatic activity was stopped by adding 2 ml of SD inhibitor [L-15 media supplemented with 0.52 mg/ml soybean trypsin inhibitor, 3.0 mg/ml BSA, and 0.04 mg/ml DNase (all Sigma)] per 1 ml HBSS. The cords were then triturated, centrifuged at 196 rcf for 5 mins, resuspended in plating medium [50% DMEM, 25% horse serum, 25% Hank’s Balanced Salt Solution (HBSS)] and plated onto the aforementioned neurosphere-derived astrocytes at a density of 150,000 cells per coverslip. Coverslips were contained in 35 mm Petri dishes, three 13 mm coverslips per dish, and placed in an incubator for 2 h. Cells were topped up with 450 μl of plating medium and 600 μl of differentiation medium [DMEM (4.5 g/ml glucose), 10 ng/ml biotin, 0.5% N1 supplement, 50 nM hydrocortisone, and 0.1 μg/ml insulin (all from Sigma)]. Cells were fed every 2–3 days by removing 500 μl of old media and replacing with 600 μl of fresh differentiation media. From DIV13, cells were fed with differentiation media without added insulin (DM^−^).

### Myelinated spinal cord cultures from mouse

Myelinating spinal cord cultures were generated from embryos of a number of mouse strains including; C57/Bl6 mice (Jackson Laboratories), *Ifnar1*^+/+^ and *Ifnar1*^−/−^ mice on a 129S7/SvEvBrdBklHprtb-m2 background (B&K Universal) and *Cst*^+/+^, *Cst*^+/−^ and Cst^−/−^ mice on a C57/Bl6 background (Prof. Hugh Willison). Mice were time-mated and pregnant females were killed by CO_2_ overdose at E13.5. Spinal cords were extracted from embryos and processed as per rat myelinating culture protocol with some amendments. Cords were enzymatically digested with 2.5% trypsin (100 μl/ml) for 15mins at 37 °C. Enzymatic activity was stopped by adding 2 ml SD inhibitor. Cords were triturated, centrifuged at 181rcf and resuspended in plating medium. Cells were plated onto 13 mm diameter glass coverslips coated with poly-L-lysine (0.1 mg/ml in boric acid buffer, pH 8.4) at a density of 165,000 cells per coverslip. Coverslips were contained in 35 mm Petri dishes, 3 coverslips per dish, and incubated for a minimum of 4 h to attach. Once visibly attached, dishes were topped up with 300 μl plating media and 600 μl differentiation media. Cells were fed as per the rat culture protocol.

### Antibody production

Once proliferating at a stable rate, cells were transferred to a CELLine cell culture flask (BD Biosciences). Cells were seeded in ultra-low endotoxin media [OptiMem supplemented with 10% ultra-low endotoxin FBS (VWR) and 1% Pen/Strep] at a density of 1.5 × 10^6^ cells/ml and maintained at a density of maximum 1.5 × 10^6^ cells/ml, supernatants collected and stored at − 20 °C until antibody purification. O4 and A4CD antibodies were purified under sterile conditions from supernatants using Hi-Trap IgM purification HP columns (GE Healthcare) as per manufacturer’s instructions. All buffers were made using sterile ultra-low endotoxin reagents. Eluted protein was dialysed in a Spectra/Por 6 Regenerated Cellulose Dialysis Membrane (Spectrum Labs) against endotoxin tested Dulbecco’s phosphate buffered saline (Invitrogen) for 24 h at 4 °C. Final antibody concentration was determined by Nanodrop (Denovix DS-11) and solution diluted to 1 mg/ml in DPBS.

### Antibody treatment

Cultures were treated at day in vitro 24 (DIV24). To treat cultures, 500 μl of media was removed and 500 μl of treatment added. Treatments were diluted in differentiation media. All antibodies were used at a final concentration of 20 μg/ml. Antibodies used in treatments included; IgM from mouse myeloma (Sigma), A4CD, O4, O1 (R&D Systems or in-house produced), A2B5 (Abcam), rhIgM22, shIgM22, rhIgM12, shIgM12, shIgM42 and shIgM201 (kindly provided by MR and AW). Untreated controls were given differentiation media alone. For IFN-β neutralisation, DIV24 rat cultures were treated with media alone, A4CD or O4 in combination with either rabbit anti-rat IFN-β neutralising antibody or normal rabbit IgG control (10 μg/ml, R&D Systems) for 24 h.

### Virus infections

BUNVGc-eGFP is a recombinant BUNV, in which the N-terminal 326 amino acid of the viral membrane Gc glycoprotein was replaced by eGFP [13]. In short, BSR-T7/5 cells were transfected with 1 μg pT7riboBUNL(+), pT7riboBUNS(+), and pT7riboBUNMGc-eGFP. After 5 h, growth media [Glasgow Minimum Essential Medium supplemented with 10% tryptose phosphate broth, 10% FBS, and (Gibco) and 1 mg/ml Geneticin (G418) sulfate (Calbiochem)]. Cells were incubated for 5–11 days at 33 °C until cytopathic effect was observed. Supernatant was clarified and stored at − 70 °C until use.

For all eGFP-Semliki Forest Virus (SFV) experiments we used an eGFP expressing variant of the neurovirulent strain SFV4. The eGFP marker gene was inserted in nsP3 via a naturally occurring XhoI site, resulting in plasmid pCMV-SFV4(Xho-EGFP)4 which was generated and kindly provided by prof Andres Merits (University of Tartu). The backbone of this plasmid has been previously described [14], further details are available from the Lead Author. Virus stocks were generated by electroporating this plasmid in BHK cells and propagated in Glasgow Minimum Essential Medium (Gibco) with 5% FBS, 10% tryptose phosphate broth (Gibco), 1% Pen/Strep at 37 °C in 5% CO_2_. When severe cytopathic effect was observed, virus-containing supernatant was centrifuged 500×g for 30 min to remove cellular debris and cell-free virus stored in small aliquots at − 70 °C. Stocks were titrated by plaque assay as described below.

DIV24 cultures were treated with either media alone, A4CD or O4 for 24 h. All media was removed and cells inoculated with 0.75% BSA (Sigma) in PBS alone or with BUNV (MOI 1) for 1 h at 5% CO_2_, shaking gently every 15 min or SFV (MOI 1) for 1 h at room temperature on a plate shaker. Inoculum was removed and fresh differentiation media was added. Cells were then incubated for a further 6–24 h, at which point supernatant was collected for plaque assay, cells washed with 0.75% BSA-PBS and prepared for further analysis.

To quantify virus stocks and production of BUNV, Vero E6 cell monolayers were incubated with serially diluted supernatant for 1 h at 37 °C then covered with 0.6% Avicel (FMC Biopolymer)-minimum essential medium overlay medium supplemented with 2% FBS. Cells were incubated for 4 days and fixed with 4% formaldehyde-PBS and stained with 0.5% (w/v) methyl violet. Virus titres were calculated and presented as PFU/ml. A similar assay was used for SFV, with the following adaptations: BHK cells were infected and maintained as described, and at 2 days post infection fixed in 10% formaldehyde-PBS and stained with 0.1% toluidine blue (Sigma).

### Drug treatment of cultures

To deplete microglia, rat cultures were treated from DIV18–28 with PLX3397 (Selleckchem) or an equivalent volume of dimethyl sulfoxide (DMSO, Sigma) every 2–3 days. Final concentration of PLX3397 in cell culture dish was maintained at 1 μM and volume of DMSO maintained at 0.1% of total media volume. For signalling pathway inhibition, DIV24 rat cultures were treated 6 h with media alone, A4CD or O4 with simultaneous administration of either DMSO or 10 μM of one of the following inhibitors; ST2825 (MedChemExpress), RU.521 (Invivogen), C-176 (Biovision) and BX795 (Invivogen).

### Fluorescence in situ hybridisation (FISH)

FISH was performed on DIV24 rat myelinating cultures treated 24 h with either A4CD or O4 using the ViewRNA Cell Plus Assay kit (Invitrogen) as per manufacturer’s instructions. First, immunocytochemistry was performed against the following antigens; GFAP (1:200, Sigma), NeuN (1:400, Millipore), Olig2 (1:500, Millipore) and Iba1 (1:500, Wako), using secondary antibodies AlexaFluor488 goat anti-rabbit IgG and AlexaFluor568 goat anti-mouse IgG1 (both Invitrogen). ViewRNA cell plus type 6 probe sets against the following genes were used; *Cxcl10*, *Mx1*, *Rsad2* and *Oasl*. Coverslips were mounted onto glass slides using Mowiol 4–88 mounting medium. Mounted slides were stored overnight at 4 °C protected from light and imaged.

### RNA isolation

RNA was extracted from rat cultures using the RNeasy Plus Micro kit (Qiagen) as per manufacturer’s instructions. RNA was extracted from mouse and virally infected cultures by removing all media from cells, adding 1 ml TRIzol® Reagent per dish and incubating for 10 min. Lysates were transferred to RNase-free tubes (Invitrogen) and stored at − 80 °C until use. RNA was isolated using the PureLink™ RNA Mini Kit (Invitrogen) as per manufacturer’s instructions. Concentration and quality of RNA was determined by Nanodrop.

### Microarray

RNA from treated DIV24 rat myelinating cultures (mock, 20 μg/ml O4 or IgM isotype control for 24 h) was quality checked with the Agilent Bioanalyzer 6000 Nano LabChip platform and biotin labelled using Ambion WT Expression Kit. The labelled RNA was then hybridized to Affymetrix GeneChip Rat Gene 2.1 ST Arrays according to manufaturer’s instructions using the Fluidics Station 450 and scanned on Gene Array Scanner 3000-7G. Each treatment group (untreated Control, O4 and IgM treatment) were set up in three replicates, analysed in Partek Genomics Suite (version 6.6, Partek) and deposited in Gene Expression Omnibus database (https://www.ncbi.nlm.nih.gov/geo/) under accession number GSE150331. Probe set level data were normalized using GC-RMA method and One-Step Tukey’s Biweight method was used to summarize to transcript cluster level. Differential expression was then calculated by one-way ANOVA comparing O4-treatment vs Control, O4-treatment vs IgM-treatment and IgM-treatment vs Control. Differentially expressed genes (fold-change > ± 1.4, FDR-adjusted *p*-value < 0.05) were then analysed for enriched KEGG pathways using Partek Pathway and for the presence of interferon regulated genes (IRGs) using the interferome database (v2.01; http://www.interferome.org/interferome/home.jspx) [15].

### cDNA synthesis and quantitative real-time PCR

Primer 3 software (http://biotools.umassmed.edu/bioapps/primer3_www.cgi) [16] was used to find suitable primer sequences on mRNA sequences from the NCBI nucleotide data base, and checked for specificity using BLAST (http://blast.ncbi.nlm.nih.gov/Blast.cgi). cDNA was synthesized from a maximum of 1 μg RNA using a QuantiTect® Reverse Transcription Kit (Qiagen) following the manufacturer’s instructions using a Biometra T3 Thermocycler (Thermofisher). Synthesised cDNA was diluted to appropriate volume using RNase-free water (1:200 for 1 μg starting RNA). Quantitative real-time PCR was performed in MicroAmp Fast Optical 96-well reaction plates (0.1 ml) (Invitrogen) with each sample being run in triplicate. Reagents per well were as follows; 7.5 μl Power SYBR™ Green PCR Master Mix (Applied Biosystems), 5.2ul RNase-free water, 0.3ul primer mix (Integrated DNA Technologies, 50 μM/primer) and 2 μl cDNA. Plates were run in an Applied Biosystems Fast Real-Time PCR System (ABI 7500) and quantified using the comparative CT (ΔΔCT) method or, for human IgM data alone, standard curve method. Cycle settings were as follows; 50 °C for 5 min, 95 °C for 10 min, followed by 40 cycles of 95 °C for 15 s, 60 °C for 1 min, and a final dissociation step at 95 °C for 15 s. *18S* was used as the housekeeping gene for mouse experiments and virus experiments. *Gapdh* was used as the housekeeping gene for all other rat experiments.

### Immunocytochemistry

Cultures were fixed with 4% formaldehyde-2% sucrose in PBS for 10 min. Fixative was replaced by 0.75% BSA-PBS, and cultures stored at 4 °C until immunocytochemistry was performed. Fixed cells were permeabilised with 0.5% Triton X for 10 min, washed with PBS, blocked with blocking buffer [1% BSA, 10% horse serum in PBS] for 45 min, incubated with primary antibody diluted in blocking buffer for 45 min, washed with PBS and incubated in dark with secondary antibody diluted in blocking buffer for 15 min. Coverslips were then washed in PBS followed by dH_2_O and mounted onto glass slides with Mowiol 4–88 mounting medium [33% w/v Mowiol® 4–88, 13.2% w/v glycerol (both Sigma), 0.05% v/v DAPI (Invitrogen) in 0.13 M Tris pH 8.5]. Primary antibodies against the following proteins were used; BUN virions (1:500, Elliott lab), NeuN (1:400, Millipore), Nestin (1:200, Millipore), GFAP (1:200, Sigma), Olig2 (Millipore, 1:200), ED1 (1:100, Abcam), Iba1 (1:500, Wako), A4CD, O4 (both 20 μg/ml, both Linington Lab), O1 (20μg/ml, R&D Systems) and A2B5 (20 μg/ml, Abcam). All secondary antibodies were purchased from Invitrogen and used at 1:400 including; AlexaFluor488 goat anti-rabbit IgG, AlexaFluor488 goat anti-mouse IgM, AlexaFluor568 goat anti-mouse IgG1 and AlexaFluor568 goat anti-mouse IgG2a. For live-staining of lipid-specific IgM, live cells were incubated with antibody (20 μg/ml, 30 min, 4 °C) and then fixed with 4% PFA. Protocol continues as above.

### Image capture and analysis

All imaging and quantification was performed blind. Coverslips from microglia depletion experiments and BUNV infections were imaged on an Olympus BX51 microscope (Olympus Lifescience) using a Retiga R6 camera and Ocular 2.0 software (both Teledyne Qiamging). Ten images were taken per coverslip, 3 coverslips per condition for every biological replicate. Images were saved as 16 bit tif files and converted to 8 bit png files using CellProfiler [17] pipeline “Ocular.cpproj”. Total dapi for each png image was quantified using CellProfiler pipeline “dapi mono.cp”. Both pipelines can be found at https://github.com/muecs/cp/tree/v1.1. Iba1-positive cells and BUNV-positive cells were counted manually using cell counter plugin (https://imagej.nih.gov/ij/plugins/cell-counter.html) with ImageJ [18]. Co-localisation of BUNV-positive dapi with other cell markers was also quantified using the cell counter plugin.

Coverslips from FISH experiments were imaged using a Zeiss Axio Imager 2 and Zen 2012 (blue edition) software. To quantify total dapi, images were saved as png files using Zen software and processed using the “dapi mono.cp” CellProfiler pipeline. Cells positive for mRNA of interest were quantified manually using the cell counter plugin in Fiji [19]. Co-localisation of mRNA-positive dapi with other cell markers was also quantified using the cell counter plugin.

### Statistical analysis

Statistical details of experiments including statistical tests used, n values and what n represents, definition of centre, dispersion and precision measures are reported in the figure legends. Statistical significance was determined using one of the following tests depending on number of samples and variables; two-tailed t-test for data with one variable and two conditions, one-way ANOVA with Tukey’s post hoc test for data with one variable and greater than two conditions, and two-way ANOVA with Bonferroni post hoc test for data with two variables and two conditions. Data were deemed significant when *p*-value was less than 0.05 and are denoted in figures as *p < 0.05, **p < 0.01, ***p < 0.001. Statistical analysis was performed in GraphPad Prism 8.

### Data availability

The microarray data generated for this study are available at the GEO repository under the following accession number GSE150331 and as supplemental information. CellProfiler pipelines can be found at https://github.com/muecs/cp/tree/v1.1

All other data supporting the findings of this study are available in the article, the Supplementary information files, or upon request to the authors. We are happy to provide a source table.

## Results

### Lipid-specific IgM provides protection against neurotropic viruses in an IFNAR1 -dependent manner

To test the hypothesis that lipid-specific IgMs induce a functional anti-viral response in the CNS, we examined the anti-viral properties of the murine IgM monoclonal antibody (mAb) O4, recognising sulfatide (3-O-sulfogalactosylceramide), a major target of the intrathecal antibody response in MS [20]. The antiviral properties of this antibody were tested in rodent myelinated cultures, which replicate much of the cellular and functional complexity of the nervous system in vivo in a tractable in vitro model [21].

To mimic the situation in MS patients who have intrathecal lipid-specific IgM in their CNS before JCV disseminates to the CNS, rat myelinated cultures were pre-treated for 24 h with O4 or isotype control A4CD, an IgM mAb specific to myelin oligodendrocyte glycoprotein (MOG) peptide. Both were purified in our laboratory under the same conditions. As JCV is an obligatory human pathogen, we used BUNV as an encephalitic model virus to infect the pre-treated cultures. BUNV, a prototype for both the *orthobunyavirus* genus and *Peribunyaviridae* family, is a tripartite negative sense single-stranded enveloped RNA virus [22] and can induce severe encephalitis in domestic animals [23].

**Fig. 1:**
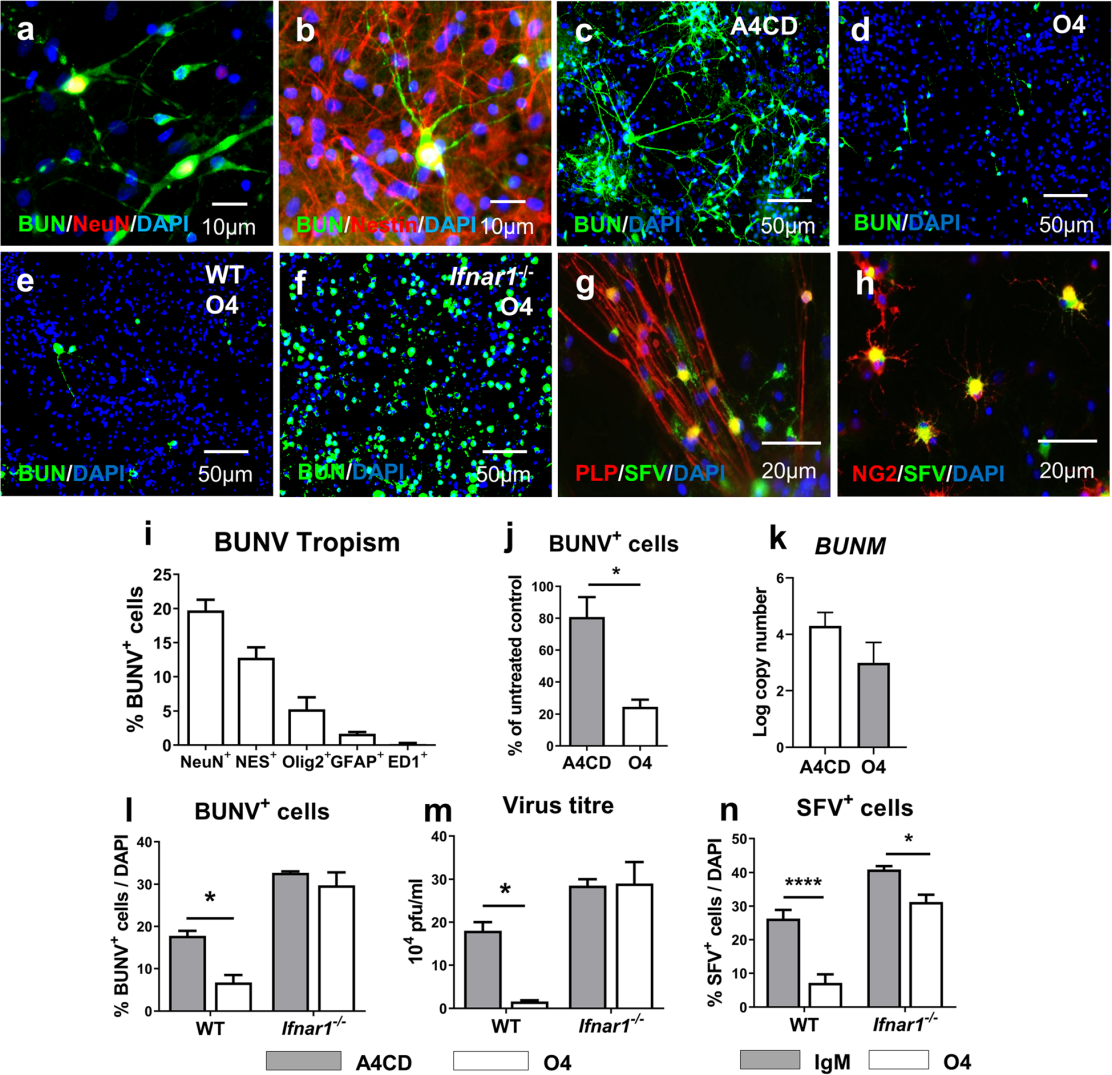
Lipid-specific IgM provides protection against neurotropic viruses in an IFNAR1-dependent manner. **a**-**d, i**-**k** Rat cultures pre-treated 24 h with A4CD or O4 and infected with BUNV (MOI = 1). **a**, **b** BUNV-infected mature neurons and neuronal progenitor. **c**, **d** A4CD and O4-treated cultures infected with BUNV. **i** BUNV tropism. **j**, **k** Immunocytochemical and RT-qPCR analysis. Data presented as mean ± SEM and analysed by paired two-tailed *t*-test. **j** *n* = 4 **k** *n* = 3. **e**, **f**, **l**, **m** WT and *Ifnar1*^−/−^ mouse cultures treated and infected as above. **e**, **f** BUNV-infected WT and *Ifnar1*^−/−^ cultures pre-treated with O4. **l**, **m** Immunocytochemical and plaque assay analysis. Data presented as mean ± SEM, analysed by two-way ANOVA and significant difference determined by Sidak’s *post-hoc* test. **l** WT *n* = 2*, Ifnar1*^−/−^ *n* = 3 **m** n = 2 for all conditions. **g**, **h**, **n** WT and *Ifnar1*^−/−^ mouse cultures treated as above and infected with SFV. **g**, **h** SFV-infected mature oligodendrocytes and oligodendrocyte progenitors. **n** Immunocytochemical analysis. Data presented as mean ± SEM, analysed by two-way ANOVA and significant difference determined by Sidak’s *post-hoc* test. *n* = 4 for all conditions. Significant differences denoted as *p < 0.05 and ****p < 0.0001

As the cellular tropism of BUNV in rodents is unknown this was first mapped by immune fluorescence microscopy using a GFP-tagged BUNV (BUNVGc-eGFP) [13] and a panel of cell type specific markers to reveal that BUNV preferentially infected cells of the neuronal lineage in myelinating cultures (Fig. 1a, b & i). Pre-treatment with O4 had a profound antiviral effect as demonstrated by quantifying BUNVGc-eGFP infected cells by immune fluorescence microscopy (*p* < 0.05) (Fig. 1c, d & j). This finding was corroborated when assessing BUNV RNA expression by RT-PCR, where a > 10-fold decrease was observed in *BUNM* transcript in O4-treated cultures (*p* = 0.1303) (Fig. 1k).

Considering our myelinated cultures lack adaptive immune cells, we hypothesised that this antiviral effect might be conferred by the innate antiviral type-I IFN pathway. To investigate this hypothesis, experiments were repeated in myelinated cultures derived from *Ifnar1*^*−/−*^ mice and wild type controls, resulting in a significant decrease in BUNVGc-eGFP infected cells and supernatant viral titre in O4-treated wild type (WT) cultures only, highlighting the dependency of this response on the type-I IFN pathway (Fig. 1e, f, l & m). Moreover, this also demonstrates the protective effect of O4 is not species-specific as it induced a similar level of antiviral activity to that observed in rat cultures.

To investigate whether this protection can be extended to other viruses with a different cellular tropism and generating different Pattern Associated Molecular Patterns (PAMPs), we investigated the antiviral potential of O4 using SFV. SFV is an encephalitogenic positive-sense single-stranded RNA virus that preferentially infects cells of the oligodendroglial lineage [24] (Fig. 1g & h) (construct kindly provided by Andres Merits). As observed for BUNV, pre-treatment with O4 inhibited replication of SFV in WT cultures (*p* < 0.001), but in this case a residual protective effect was observed in *Ifnar1*^*−/−*^ cultures (*p* < 0.05) (Fig. 1n). This may be due to steric hindrance of antibody that is bound to the oligodendrocyte cell surface restricting viral access and/or disruption of receptor-mediated endocytosis but nonetheless, the dominant protective effect is largely IFNAR1-dependent.

Together these results demonstrate that the lipid-specific IgM mAb O4 exhibits antiviral properties that can inhibit virus replication in neurons and glia in an IFNAR1*-*dependent manner. This supports the idea that intrathecal lipid-specific IgM found in MS patients may contribute to protection against JCV and limit risk of PML.

### Lipid-specific IgM induces an antiviral transcriptional signature in vitro

**Fig. 2:**
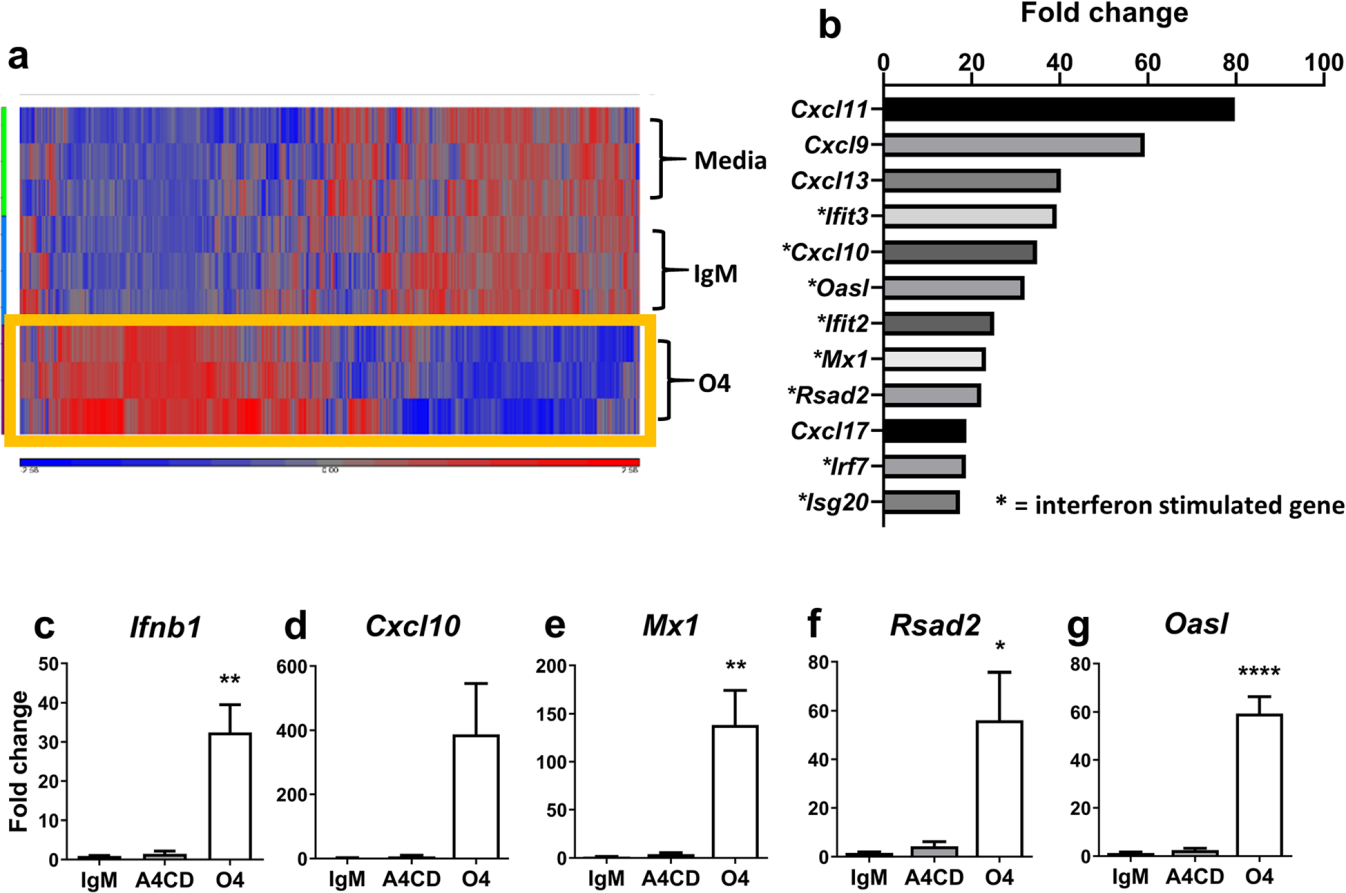
Lipid-specific IgM induces anti-viral transcriptional signature in vitro. **a**, **b** Microarray analysis of rat myelinating cultures treated 24 h with media alone, IgM from mouse myeloma (IgM, Sigma) or O4. **a** Cluster analysis of all significant genes (FDR *p*-value < 0.05); heat map shows standardized gene expression level of each gene with high expression in red and low expression in blue. **b** Bar graph showing genes most upregulated by O4, interferon stimulated genes denoted by an asterisk. **c**-**g** RT-qPCR analysis of rat cultures treated with media alone, IgM, A4CD or O4 for **c** 4 h and **d**, **g** 24 h. Data analysed by one-way ANOVA and significance determined by Tukey’s post hoc test. IgM (*n* = 4), A4CD (*n* = 3) and O4 (*n* = 4). Significant differences denoted as *p < 0.05, **p < 0.01 and ****p < 0.0001

To gain further insight into the mechanistic basis of this antiviral response, a microarray was performed on cultures treated for 24 h with O4, IgM isotype control or media alone. O4 differentially regulated 543 transcripts compared to the IgM control (431 upregulated, 112 down regulated; fold-change ≥ ±1.4; FDR-adjusted *p* ≤ 0.05), whilst no significant differences were observed between untreated and IgM control-treated cultures (Fig. 2a, GSE150331).

The transcriptional response induced by mAb O4 was characterised by increased expression of multiple genes that play important roles in the development of antiviral responses in the CNS (Fig. 2b and Table S1). These include chemokines (chemotactic cytokines) *Cxcl10* and *Ccl5*, the products of which co-ordinate recruitment of immune effector cells into the CNS, [25], as well as numerous genes such as *Oasl*, *Mx1* and *Rsad2*, which encode proteins that restrict viral replication [26]. It is therefore not surprising that pathway enrichment analysis identified viral diseases as the most significantly enriched human disease pathways associated with this response (Table S2). Moreover, the other most significantly enriched organismal system pathways included NOD-like, Toll-like and RIG1-like signaling pathways (Table S3). These observations led us to speculate the IFN-like response triggered by O4 involves activation of one or more Pattern Recognition Receptor (PRR)-dependent pathways; a concept supported by the demonstration that as much as 72% of all differentially regulated genes are interferon-stimulated genes (ISGs) based on the murine interferome database (Fig. 2b and Table S1) [15].

To validate these microarray data, we selected IFN-β and several top-ranking ISGs for RT-qPCR analysis. *Ifnb1* was rapidly and significantly upregulated within 4 h of O4 treatment (*p* < 0.01) (Fig. 2c). After 24 h, we observed a significant upregulation of the chemokine genes *Cxcl10* (*p* = 0.0552) and *Ccl5* (*p* < 0.05), and the antiviral transcripts *Oasl* (*p* < 0.0001), *Mx1* (*p* < 0.01), *Rsad2* (*p* < 0.05), *Ifit2* (*p* < 0.01), *Isg15* (*p* < 0.01), and *Isg20* (*p* < 0.05) (Fig. 2d-g and Suppl. Fig. 1A-D) compared to the IgM and untreated controls.

In summary, these data demonstrate that O4 induces an “IFN-like” response in CNS cells, explaining the potent antiviral effect of O4 against two genetically distinct neurotropic model viruses.

### The response induced by O4 is IFN-β- and IFNAR-dependent

To investigate the importance of IFN signalling for this response, we first mapped the kinetics of IFN and ISG expression induced by O4. Expression of *Ifnb1* was detected after 4 h, peaked at 8 h and then declined slowly over the following 10 h (Suppl. Fig. 2A). In contrast, ISG expression increased markedly between 8 and 18 h post-treatment (Suppl. Fig. 2B-H). Induction of *Ifnb1* and these selected ISGs was not observed in cultures treated with control IgM (Fig. 2c-g).

**Fig. 3:**
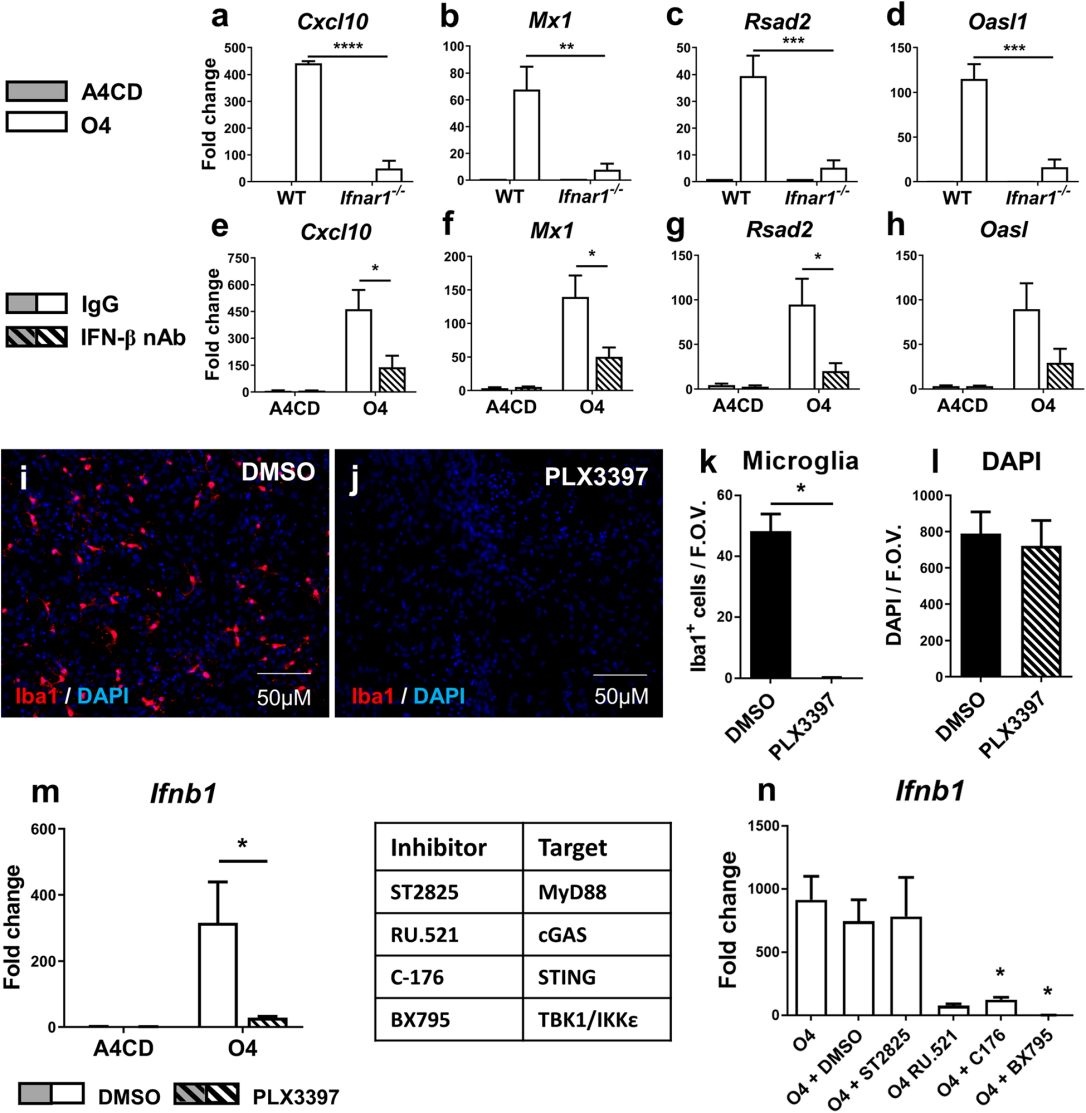
O4 response is mediated by *Ifnar1* and microglia-derived interferon-β in a cGAS-STING-TBK1-IKKε dependent manner. **a**-**h** RT-qPCR analysis of ISG expression in **a**-**d** WT and *Ifnar1*^−/−^ mouse cultures treated 24 h with A4CD or O4 and **e**-**h** rat cultures treated 24 h with A4CD or O4 in combination with rabbit IgG or anti-rat IFN-β neutralising antibody. Data presented as mean fold change compared to untreated control ± SEM, analysed by two-way ANOVA, significant interaction denoted *p < 0.05, **p < 0.01, ***p < 0.001 and ****p < 0.0001. WT (*n* = 2), *Ifnar1*^−/−^ (*n* = 4), IFN-β neutralisation experiment (*n* = 3 all conditions). **i-m** Rat cultures treated 10 days with DMSO or PLX3397 followed by 6 h treatment with A4CD or O4. **i**, **j** Iba1 staining. **k**, **l** Iba1^+^ and DAPI^+^ cells. Data presented as mean ± SEM, significant difference determined by paired two-tailed *t*-test, *p < 0.05. **m** RT-qPCR analysis of *Ifnb1* expression. Data presented as mean fold change compared to untreated control ± SEM, analysed by two-way ANOVA, significant interaction denoted *p < 0.05, *n* = 3 for all conditions. **n** *Ifnb1* expression in rat cultures treated 6 h with O4 alone or in combination with vehicle or inhibitor of interest (10 μM). Data presented as mean fold change compared to untreated control ± SEM, analysed by one-way ANOVA with Tukey’s post hoc test. Significant difference compared to O4 alone denoted as *p < 0.05. O4 alone (*n* = 6), DMSO (*n* = 6), ST2825 (*n* = 3), RU.521 (*n* = 2), C-176 (*n* = 5) and BX795 (*n* = 3)

To assess whether the O4 response was dependent on type-I IFN signalling, WT and *Ifnar1*^*−/−*^ mouse myelinating cultures were treated with media alone, control IgM (A4CD) or O4 for 24 h. Induction of ISGs by O4 was abrogated almost completely in *Ifnar1*^*−/−*^ cultures, showing a significant interaction between the presence of Type I IFN receptors and upregulation of *Cxcl10* (*p* < 0.0001), *Mx1* (*p* < 0.01), *Rsad2* (*p* < 0.001) and *Oasl* (*p* < 0.001) (Fig. 3a-d). Induction of ISGs by O4 is therefore mediated predominantly via activation of the type-I IFN pathway.

To confirm the ISG induction was specifically IFN-β-dependent as indicated by the mRNA expression data (Suppl. Fig. 2A), rat myelinating cultures were treated for 24 h with A4CD or O4 in the presence of an IFN-β neutralising antibody or rabbit IgG as a control. O4-induced ISG expression was attenuated significantly in the presence of the IFN-β neutralising antibody with significant interaction between treatment and neutralisation being observed for mRNA expression of *Cxcl10* (*p* < 0.05), *Mx1* (*p* < 0.05) and *Rsad2* (*p* < 0.05) (Fig. 3e-g). Expression of *OasI* was also reduced when IFN-β was neutralised but this did not reach statistical significance (*p =* 0.1072) (Fig. 3h).

These data confirm O4-mediated induction of IFN-β is responsible for IFNAR1*-*dependent expression of antiviral ISGs in these CNS cultures, an observation supporting our hypothesis intrathecal synthesis of lipid-specific IgMs can have antiviral properties in the CNS.

### Microglia are the major source of *Ifnb1* in a cGAS-STING-dependent manner

To further elucidate the mechanism of action by O4, we sought to identify those cells responsible for producing IFN-β. Previous studies indicate microglia are a major source of IFN-β [27, 28] and may therefore orchestrate ISG expression in other neural cells. We therefore used the colony-stimulating factor 1 receptor (CSF-1R) inhibitor PLX3397 to deplete microglia prior to treating the cultures with O4 or control IgM [29]. PLX3397 reduced Iba-1^+^ microglia by > 99% without affecting viability of the other cell types (Fig. 3i-l). This was accompanied by an almost complete abrogation of *Ifnb1* expression in O4-treated cultures (Fig. 3m). We therefore conclude microglia are the major source of O4-induced *Ifnb1* expression in myelinating cultures.

To gain understanding as to how O4 upregulates IFN-β in microglia, we screened a panel of inhibitors that act upstream of known IFN-β-inducing pathways. Rat myelinated cultures were treated for 6 h with O4 combined with vehicle or inhibitor of interest (10 μM). *Ifnb1* expression was substantially depleted in cultures where cGAS (RU.521) or STING (C-176, *p* < 0.05) were inhibited, and completely ablated by inhibition of TBK1/IKKε (BX795, *p* < 0.05) (Fig. 3n). This would suggest that *Ifnb1* after O4 treatment is upregulated predominantly by a cGAS-STING-TBK1/IKKε dependent pathway.

### O4 induces cell type specific patterns of ISG expression in the CNS

**Fig. 4:**
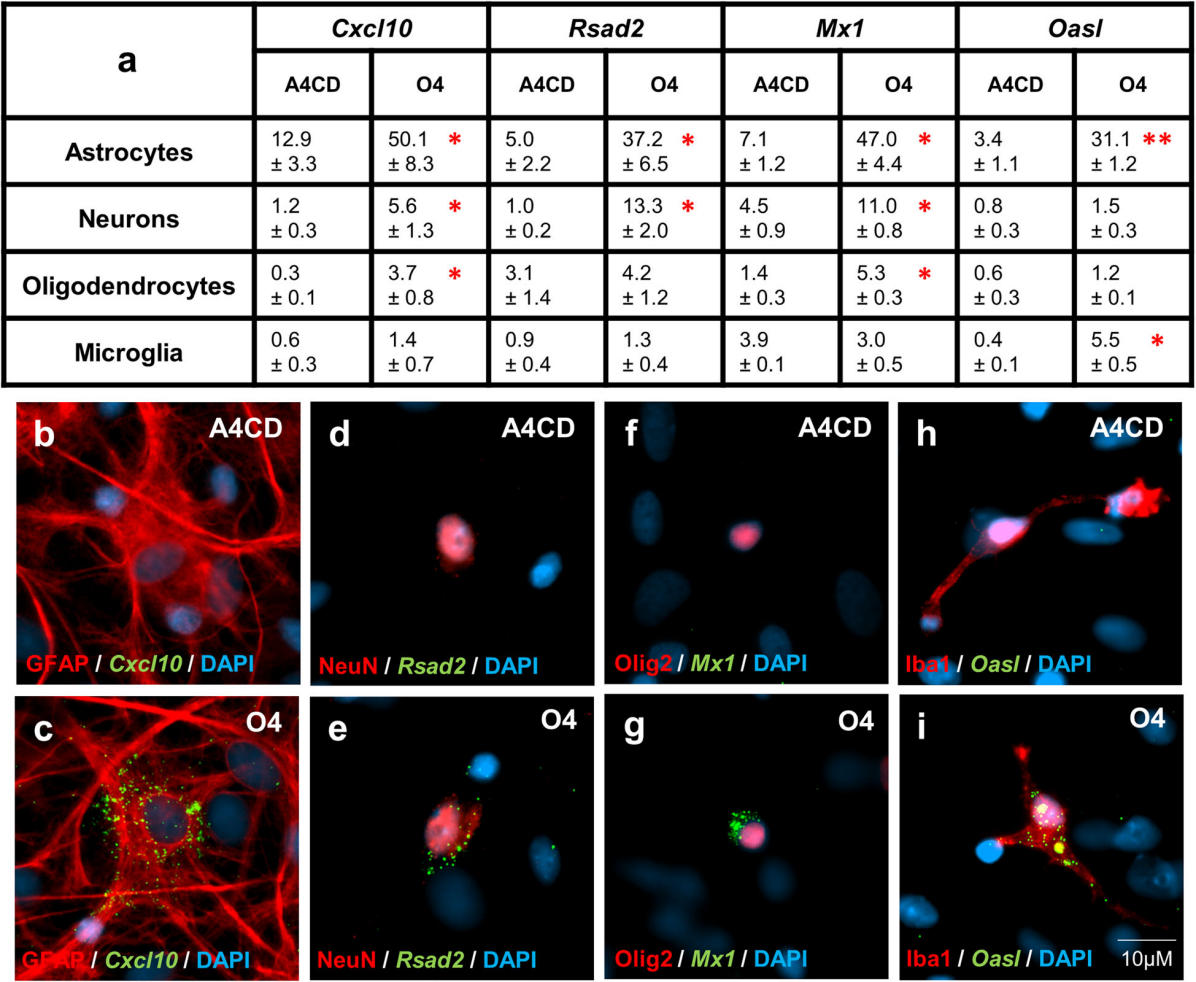
O4 induces cell type specific patterns of ISG expression in the CNS. **a**-**i** DIV24 rat cultures treated 24 h with A4CD or O4. **a** Table showing differential cellular expression of ISGs. Data analysed by paired two-tailed *t-*test. Significant differences denoted by *p < 0.05 and **p < 0.01. *n* = 3. **b**, **c** GFAP (astrocytes) and *Cxcl10* in A4CD and O4-treated cultures respectively. **(d, e)** NeuN (neurons) and *Rsad2* in A4CD and O4-treated cultures respectively. **f**, **g** Olig2 (oligodendrocytes) and *Mx1* in A4CD and O4-treated cultures respectively. **h**, **i** Iba1 (microglia) and *Oasl* in A4CD and O4-treated cultures respectively

Having identified microglia as the major source of IFN-β, we next asked if IFN-β expression orchestrates ISG expression across all major CNS cell types. For many ISGs there are no suitable antibodies commercially available, so we combined Fluorescent In Situ Hybridisation (FISH) with cell-specific antibodies to visualise transcripts encoding candidate ISGs (*Cxcl10*, *Mx1*, *Rsad2* and *Oasl*) in neurons, oligodendroglia, astrocytes and microglia. Compared to A4CD-treated controls, O4 induced a significant increase in cells expressing these candidate ISGs (*Cxcl10 p* < 0.001; *Mx1 p* < 0.001; *Rsad2 p* < 0.001 and *Oasl p* < 0.01) (Suppl. Fig. 3) and each cell type upregulated at least one ISG (Fig. 4a). Astrocytes upregulated all four ISGs, neurons upregulated *Rsad2*, *Cxcl10* and *Mx1*, oligodendrocytes upregulated *Cxcl10* and *Mx1* whilst microglia only upregulated *Oasl* in this model of the CNS (Fig. 4)*.*

These observations reinforce our hypothesis that intrathecal synthesis of lipid-specific IgM protects MS patients from natalizumab-associated PML and presumably other viral infections of the CNS.

### Lipid-specific IgM-mediated antiviral responses are not sulfatide dependent

**Fig. 5:**
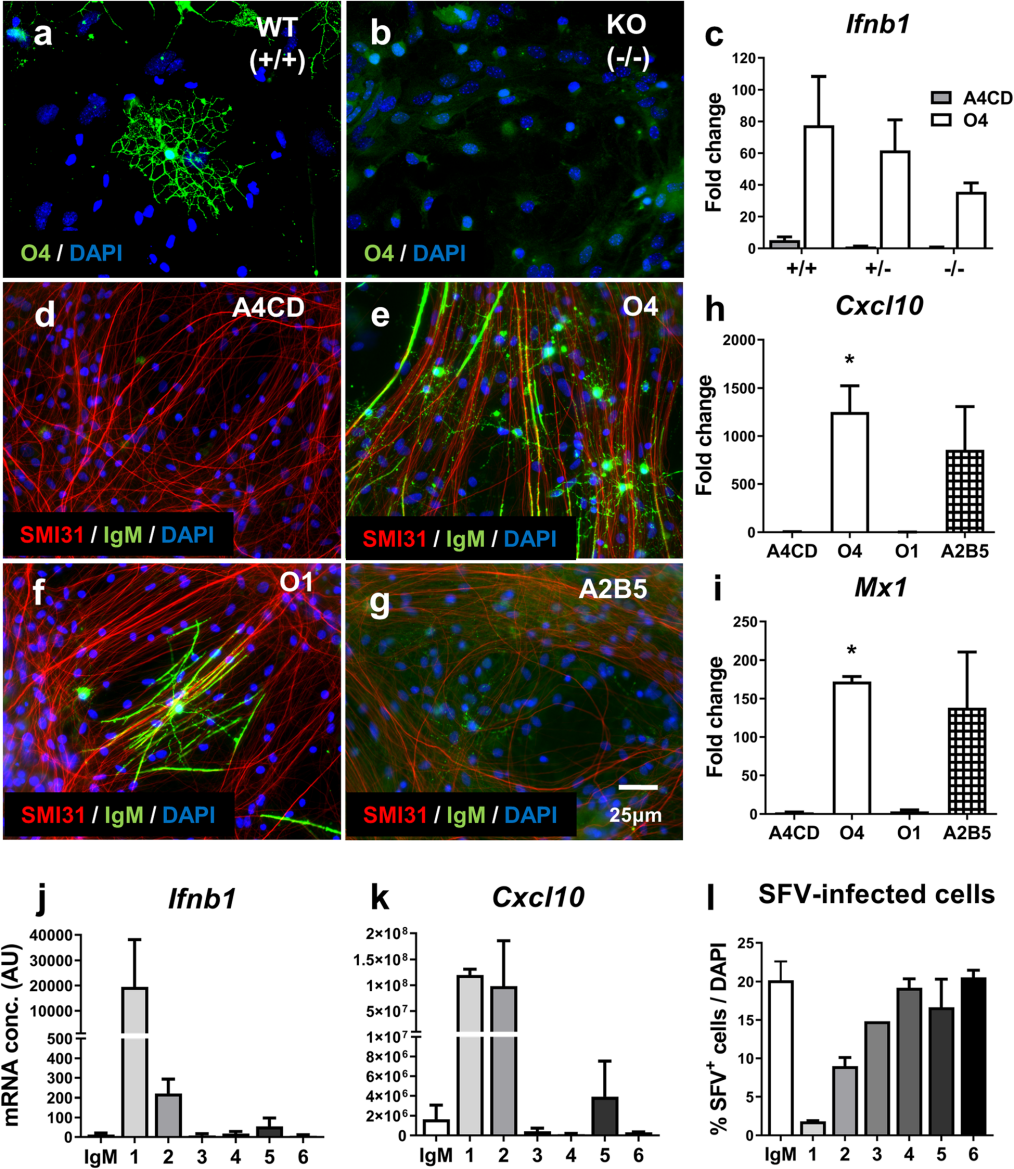
Lipid-specific IgM mediated antiviral responses are not sulfatide-dependent and identification of similar antibodies in human repertoire. **a**, **b** O4 staining in WT and *Cst*^*−/−*^ mouse cultures respectively. **c** *Ifnb1* expression in DIV24 *Cst*^*+/+*^, *Cst*^*+/−*^ and *Cst*^*−/−*^ cultures treated 6 h with A4CD or O4. Data presented as mean fold change compared to untreated control ± SEM and analysed by two-way ANOVA. **d**-**g** Representative images of binding patterns of A4CD, O4, O1 and A2B5 in DIV24 rat cultures. **h**, **i** ISG expression in DIV24 rat cultures treated 24 h with A4CD, O4, O1 and A2B5. Data presented as mean fold change compared to untreated control ± SEM and analysed by one-way ANOVA with Tukey’s post hoc test. Significant difference compared to A4CD denoted as *p < 0.05, *n* = 3 for all conditions. **j**-**l** Screening of patient-derived human IgM antibodies (hIgM’s) **j** *Ifnb1* expression 2 h post-treatment. **k** *Cxcl10* expression 24 h post-treatment. Data represent two technical replicates from one biological n (DIV24 mouse cultures) and are presented as mean ± SD. **l** Immunocytochemical analysis of DIV24 mouse cultures pre-treated 24 h with hIgM’s and infected with SFV. Data presented as mean ± SD and represent a biological n of 2 for all conditions except sample 3 (*n* = 1)

We next asked if the STING-dependent induction of IFN-β is dependent on O4 binding sulfatide; a galactosphingolipid highly enriched in myelin. O4 is poly-reactive and not only binds sulfatide but also seminolipid (3-sulfogalactosyl-1-alkyl-2-acyl-sn-glycerol) and a variety of other ligands [30]. To differentiate between these targets we used cerebroside sulfotransferase (CST) deficient mice that lack the ability to synthesise sulfatide and seminolipid [31], resulting in CST^−/−^ cells to which O4 cannot bind (Fig. 5a & b).

Comparison of cultures from CST^+/+^, CST^+/−^ and CST^−/−^ embryos revealed that specific binding of O4 to sulfatide, seminolipid or any other CST-dependent sulfoglycolipid exposed at the outer surface of myelin and oligodendrocytes was not required for O4 induction of *Ifnb* (Fig. 5a-c). Whilst O4-indued *Ifnb* expression by CST^−/−^ cultures was lower for CST^+/+^ cultures, this difference did not reach statistical significance and could be due to the lower percentage of microglia in the CST^−/−^ cultures (data not shown). Recognition of cell surface sulfogalactolipids by O4 is therefore not an absolute requirement to trigger induction of *Ifnb1*. This was unexpected as our working hypothesis was that induction of antiviral activity involved damage associated molecular patterns (DAMPs) generated in response to O4 binding to the oligodendrocyte/myelin surface.

This observation prompted us to explore whether other glycosphingolipid-reactive IgM mAbs would also induce *Ifnb1* expression in this culture system. This was investigated using O1 which is specific for galactosyl ceramide [32] and A2B5 which recognises multiple c-series gangliosides [33]. Immunofluorescence staining confirmed O1 bound extensively to oligodendroglia and myelin (Fig. 5f), whilst A2B5 weakly labelled a population of cells identified tentatively as neural progenitors (Fig. 5g). Strikingly, we found A2B5 upregulated expression of *Cxcl10* and *Mx1* to levels comparable to that in O4 treated cultures, whereas O1 had no such effect (Fig. 5h & i). These data indicate induction of antiviral activity by lipid-reactive IgM is not restricted to O4 but can be mediated by other lipid-reactive IgMs and does not require recognition of CNS myelin.

### Screening of human-derived IgM antibodies identifies candidate with antiviral properties

To determine if IgMs with antiviral activity are present in the human antibody repertoire, we investigated a small panel of human IgMs isolated from patients with IgM gammopathies such as Waldenstrom’s macroglobulinemia [34]. Using mouse myelinated cultures, we found three out of six tested antibodies induced expression of *Ifnb1* at 2 h (Fig. 5j) and *Cxcl10* at 24 h (Fig. 5k); shIgM22 (1), 2) rhIgM22 (2) which is a recombinant version of shIgM22 and to a far lesser extent shIgM42 (5). To assess whether induction of *Ifnb1* supported a functional antiviral response we pre-treated cultures with human IgMs for 24 h and then infected them with SFV. We observed a substantial decrease in the percentage of cells infected with SFV in cultures pre-treated with rhIgM22 and to a lesser extent shIgM22 (Fig. 5l); an effect that correlates with their ability to induce *Ifnb1*. Immunofluorescence staining showed these two antiviral antibodies also bind oligodendrocytes (Suppl. Fig. 4A & B) whilst the remaining antibodies in this screen target neurons (Suppl. Fig. 4C-E) or failed to bind in culture (Suppl. Fig. 4F).

Together, the data indicate a subset of lipid-specific IgM antibodies exists in mice and humans that can induce a functional antiviral response in the CNS. This previously unreported mechanism offers an explanation for the association between intrathecal lipid-specific IgM and protection against JCV in MS patients.

## Discussion

Sustained intrathecal antibody synthesis is the most consistent and well-documented immunological abnormality associated with MS; its most obvious manifestation being the presence of oligoclonal immunoglobulins in patient cerebrospinal fluid [35]. The specificity profile of this response is complex, but contains a significant component directed against lipid antigens in particular sulfatide (3-O-sulfated galactosyl ceramide) [20, 36, 37]. It has been suggested this lipid-specific antibody response plays an important role in modulating disease activity in the CNS as intrathecal synthesis of lipid-reactive IgM correlates not only with a more aggressive disease course [38], but also a reduced risk of patients developing natalizumab-associated PML [11]. Inspired by the latter report, we hypothesised a subset of lipid-reactive IgMs might enhance innate antiviral activity in the CNS. We now demonstrate that several different lipid-reactive IgM mAbs can initiate a functional antiviral response in primary myelinating cultures that reproduce the cellular and functional complexity of the CNS [21, 39]. This response is dependent on cGAS/STING-mediated upregulation of IFN-β in microglia, which then triggers IFNAR1-dependent expression of antiviral ISGs in oligodendrocytes, neurons, astrocytes and microglia.

Identification of microglia as the source of IFN-β induced by O4 concurs with studies demonstrating microglia are major producers of IFN-β in other inflammatory and viral disease models [27, 28, 40]. IFN-β subsequently upregulated expression of ISGs in surrounding neurons and glia via activation of IFNAR, which is expressed ubiquitously by cells throughout the CNS [41]. However, the response of individual cells to IFN-β will differ due to intrinsic differences in signalling thresholds and basal levels of proteins regulating the downstream response [42, 43]. Indeed, our comparative analyses of four selected ISGs that are upregulated in response to O4 indicates astrocytes are especially efficient at responding to microglia-derived IFN-β (Fig. 4a).

Astrocytes are the major glial cell type of the CNS and together with microglia are integral components of the BBB [44]. For viruses that enter the CNS by crossing the vascular endothelium, astrocytes are the first cells these viruses encounter. The robust astrocytic response to microglia-derived IFN-β may therefore represent a critical “gate keeper” function that helps protects the CNS compartment from systemic infections [45, 46]. However, our data demonstrate O4 not only initiates antiviral activity in astrocytes but also in oligodendrocytes and neurons. This is important as JCV infects all three cell types in PML [47, 48], and supports our hypothesis that a subset of lipid-reactive IgMs enhance antiviral activity in the CNS, protecting MS patients from natalizumab-associated PML. We were unable to confirm this directly as JCV is an obligate human pathogen. Nonetheless, our identification of human IgMs with similar antiviral properties, together with reports that IFN-β inhibits JCV replication in human glia [49, 50] support this concept.

In addition to providing a logical mechanism linking intrathecal synthesis of lipid-reactive IgM with protection from natalizumab-associated PML [11], our data also provide new insight into why this antibody response is also associated with a more aggressive MS disease course [38]. In addition to inducing multiple antiviral genes, O4 also upregulated expression of a large number of chemokine genes (Fig. 2b, Table S1). Chemokines are important regulators of immune cell migration and play key roles in the development of inflammatory responses in the CNS [51]. We therefore interpret this transcriptional response as a mechanism to recruit immune cells across the BBB in response to a perceived viral threat. The observed chemokine response is mainly involved in recruitment of T and B cells, and monocytes, all of which can mediate tissue damage in neuroinflammatory diseases [52–57]. This multifaceted cellular response is predicted to accelerate clearance of viral pathogens, but in the context of MS may enhance inflammatory activity in the CNS, resulting in a more aggressive disease course.

This is perhaps surprising as IFN-β is a well-recognised treatment for MS. However, we attribute this apparent dichotomy to marked differences in the biological effects of local, endogenous production of IFN-β within the CNS as opposed to those induced by systemic treatment in the periphery. Our data indicate induction of IFΝ-β by interferogenic IgM’s stimulates a co-ordinated but sequestered antiviral response in the CNS that includes a pro-inflammatory component predicted to enhance disease activity in MS. In contrast, the mechanisms responsible for the therapeutic efficacy of IFN-β in MS remain poorly understood. It is unclear if systemic treatment with IFN- β also triggers antiviral activity in the CNS, but its mode of action include effects that reduce leukocyte migration across the blood brain barrier [58–60]. This is the same mechanism by which natalizumab suppresses immune surveillance of the CNS, a major factor contributing to the risk of patients developing PML. As such, this may explain why some of the first cases of natalizumab-associated PML occurred in MS patients undergoing combinatorial treatment with IFN-β [61, 62].

Whilst PML is relatively rare, many other viruses cause life-threatening infections of the CNS, the prevalence of which is increasing due to the continuing emergence of encephalitogenic viruses [63, 64]. Moreover, in over half of presumptive cases of viral encephalitis the causative agent is never identified [65], and for most viral pathogens there are no effective antivirals available, as a consequence the clinical outcome is often poor [66]. Pan-viral therapeutic strategies that boost innate antiviral activity in the CNS rather than attempting to target the causative agent specifically may therefore transform treatment of these diseases. Activating skin macrophages at the inoculation site provides pan-viral protection against mosquito-transmitted viruses [67], suggesting a similar outcome might be achieved within the CNS by activating microglia. It would be of interest to determine whether the presence of intrathecal lipid-reactive IgM is correlated to differences in the incidence of complications due to other viruses in the nervous system. Our data obtained using BUNV and SFV as genetically unrelated, model neurotropic viruses support this concept, but there are major challenges associated with using lipid-reactive IgMs to stimulate microglial responses in the CNS.

Challenges of therapeutic use of IgMs include the potential risk of neurological deficits caused by antibodies that can bind to the myelin/oligodendrocyte surface [36, 68] and the difficulty achieving sustained high concentrations of IgM in the CNS compartment [69, 70]. Miniaturization of intrathecal pumps may overcome the latter problem, but to develop a safe and more generally accessible pharmacological treatment strategy we must understand how lipid-specific IgMs induce expression of IFN-β in the CNS.

We initially focused on the role of antibody-specificity, as this is the primary factor determining the functional activity of somatically mature antibodies. However, those IgMs inducing an antiviral effect in the CNS are close to germline and polyreactive, features indicating they are natural antibodies [71]. As yet no common reactivity was identified that would account for the ability of O4, A2B5 and rhIgM22 to induce IFN-β in the CNS. O4 recognizes sulfatide, seminolipid and other targets [30], A2B5 binds multiple c-series gangliosides [72] and rhIgM22 binds one or more unidentified ligands [73]. Notably, O4 and hIgM22 are both natural IgMs with few somatic mutations and we hypothesise their polyreactivity underlies their ability to induce IFN-β in myelinated cultures. Our finding that neither sulfogalactolipids (Fig. 5a-c) nor binding to myelin/oligodendrocytes (Fig. 5f-i) are prerequisites to trigger IFN-β expression by microglia suggest cellular specificity is not a major factor determining their activity. Based on these observations and our demonstration IFN-β induction by O4 involves activation of a cGAS-STING-TBK1/IKKε dependent pathway, we are currently exploring the hypothesis that interferogenic IgMs enhance delivery of an endogenous cGAS/STING agonist to microglia. A precedent which is provided by studies demonstrating that immune complexes can induce type I IFN expression in plasmacytoid dendritic cells by enhancing delivery of host nucleic acids [74, 75].

## Conclusion

In conclusion, we identify a logical mechanism to explain the association between intrathecal synthesis of lipid reactive IgM and decreased incidence of natalizumab-associated PML in MS. This strengthens the case for using the presence of intrathecal lipid-specific IgM for further risk stratification when deciding on treatment regimens for MS patients. Furthermore, we hope this mechanism can be exploited to guide development of pan-viral treatment strategies for viral encephalitis.

## Supplementary information

**Additional file 1: Supplementary Figure 1.** ISG expression in rat cultures treated 24 hrs with commercial IgM, A4CD and O4. **Supplementary Figure 2.** Kinetics of O4 induction of type-I interferon signalling. **Supplementary Figure 3.** Visualisation of ISG expression in rat cultures treated 24 hrs with A4CD or O4. **Supplementary Figure 4.** Binding patterns of screened human IgMs in DIV24 mouse cultures.

**Additional file 2: Table S1.** Fifty most upregulated genes upregulated in DIV24 rat myelinating cultures treated 24 hrs with O4 compared to IgM control. **Table S2.** Significantly altered human disease pathways. **Table S3.** Significantly altered organismal system pathways. **Table S4.** Primer sequences.
