# Supplementary material for: Lipid-specific IgMs induce antiviral responses in the CNS: implications for progressive multifocal leukoencephalopathy in multiple sclerosis
Source: Acta Neuropathol Commun. 2020 Aug 13;8:135. doi: 10.1186/s40478-020-01011-7 (PMC7427287; doi:10.1186/s40478-020-01011-7)
Supplement: Supplementary file 1 — Additional file 1: Supplementary Figure 1. ISG expression in rat cultures treated 24 hrs with commercial IgM, A4CD and O4. Supplementary Figure 2. Kinetics of O4 induction of type-I interferon signalling. Supplementary Figure 3. Visualisation of ISG expression in rat cultures treated 24 hrs with A4CD or O4. Supplementary Figure 4. Binding patterns of screened human IgMs in DIV24 mouse cultures. [file 40478_2020_1011_MOESM1_ESM.pptx]

## Slide 1
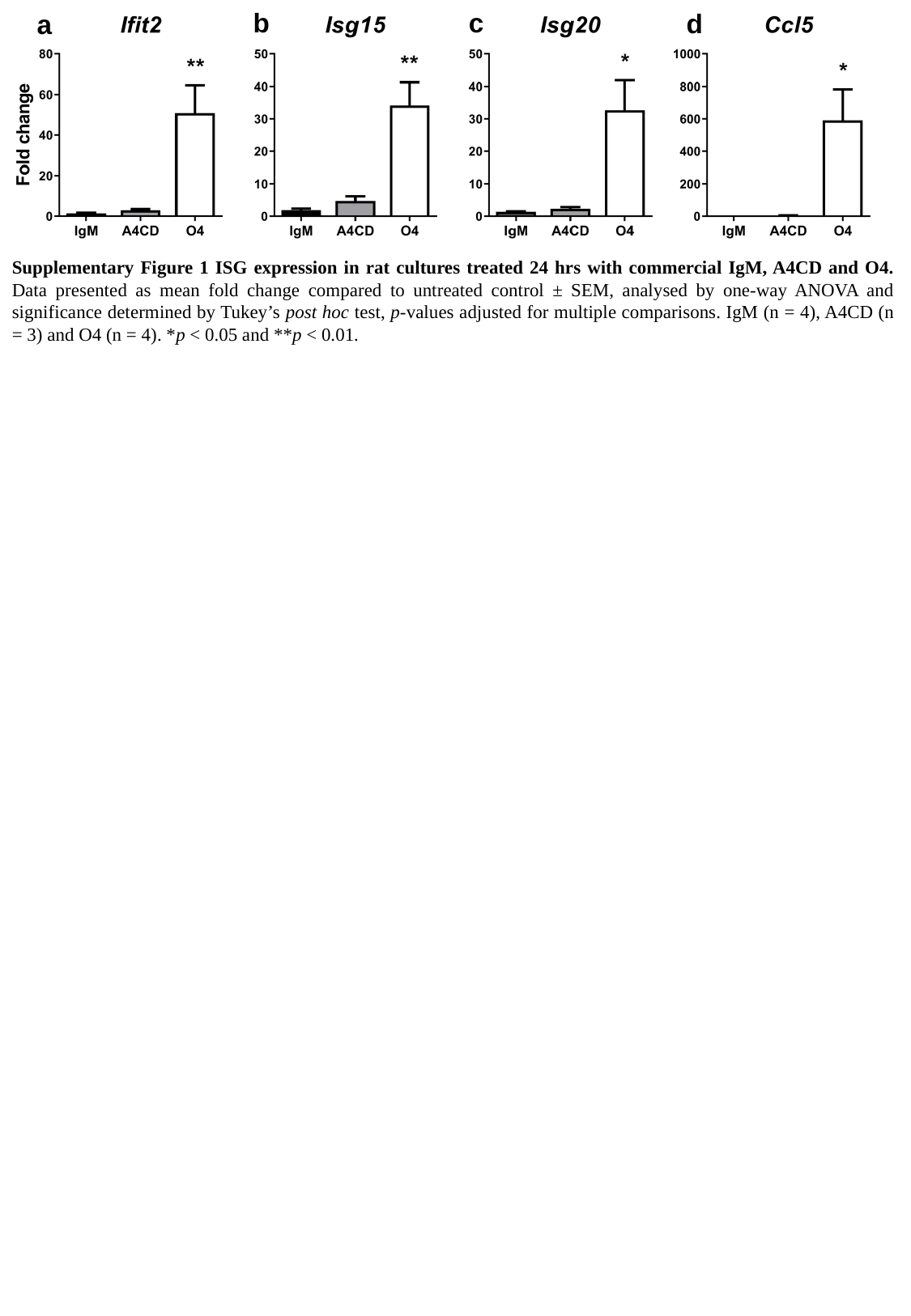

b
c
d
a
Supplementary Figure 1 ISG expression in rat cultures treated 24 hrs with commercial IgM, A4CD and O4. Data presented as mean fold change compared to untreated control ± SEM, analysed by one-way ANOVA and significance determined by Tukey’s post hoc test, p-values adjusted for multiple comparisons. IgM (n = 4), A4CD (n = 3) and O4 (n = 4). *p < 0.05 and **p < 0.01.

## Slide 2
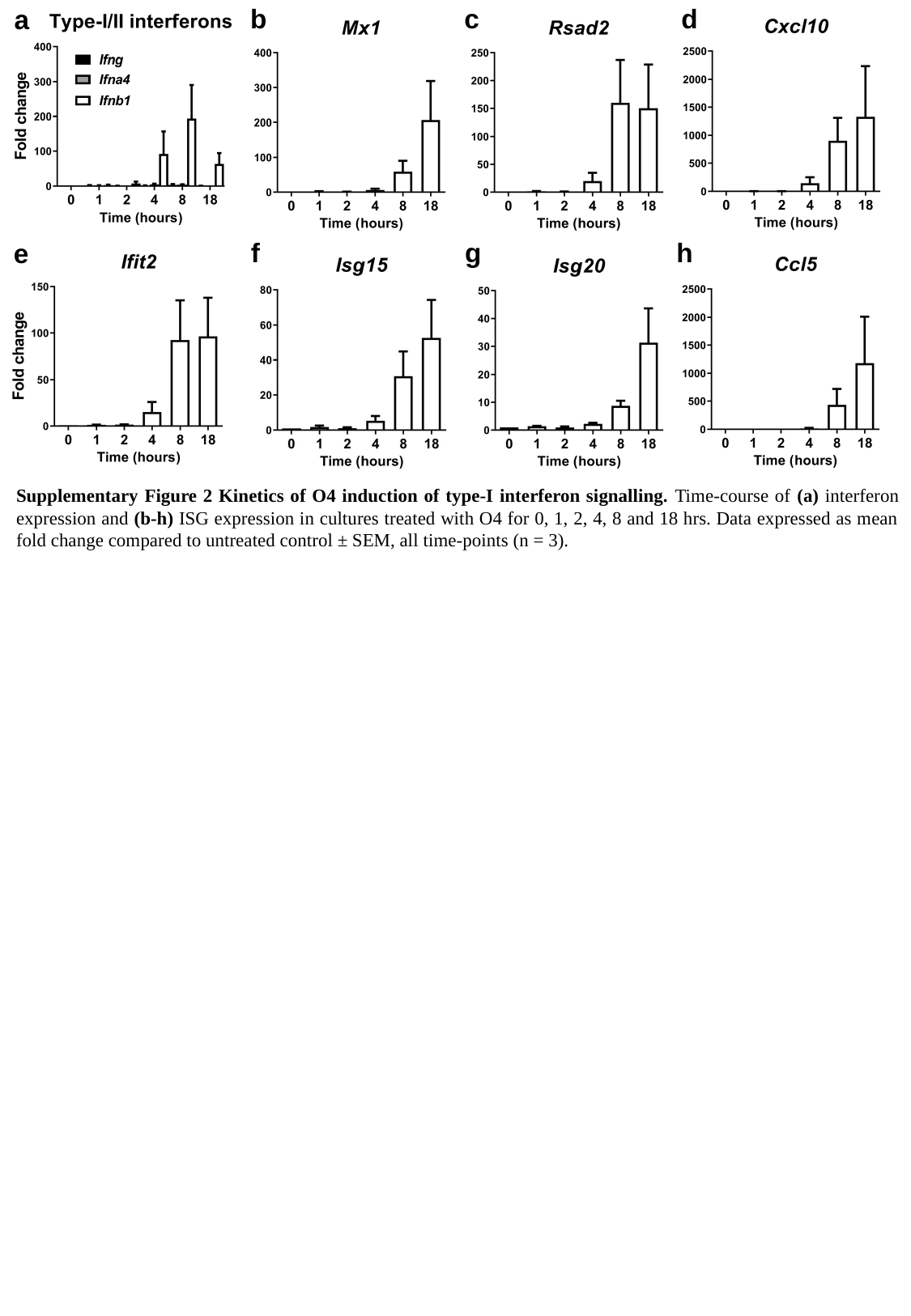

b
c
d
a
f
g
h
e
Supplementary Figure 2 Kinetics of O4 induction of type-I interferon signalling. Time-course of (a) interferon expression and (b-h) ISG expression in cultures treated with O4 for 0, 1, 2, 4, 8 and 18 hrs. Data expressed as mean fold change compared to untreated control ± SEM, all time-points (n = 3).

## Slide 3
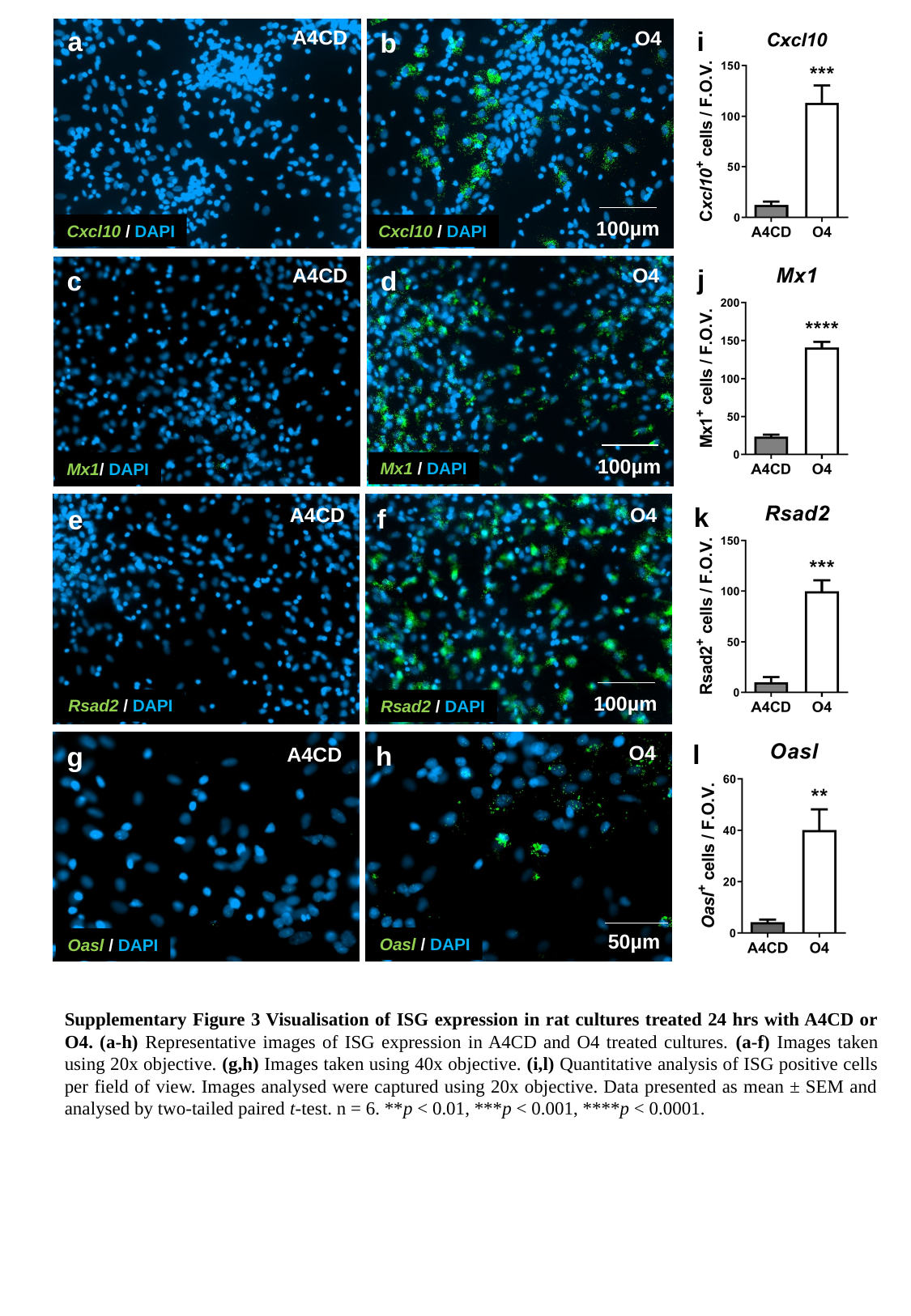

a
i
A4CD
Cxcl10 / DAPI
O4
Cxcl10 / DAPI
b
100µm
j
O4
Mx1 / DAPI
A4CD
Mx1/ DAPI
c
d
100µm
O4
Rsad2 / DAPI
A4CD
Rsad2 / DAPI
k
f
e
100µm
l
O4
Oasl / DAPI
A4CD
h
g
50µm
Oasl / DAPI
Supplementary Figure 3 Visualisation of ISG expression in rat cultures treated 24 hrs with A4CD or O4. (a-h) Representative images of ISG expression in A4CD and O4 treated cultures. (a-f) Images taken using 20x objective. (g,h) Images taken using 40x objective. (i,l) Quantitative analysis of ISG positive cells per field of view. Images analysed were captured using 20x objective. Data presented as mean ± SEM and analysed by two-tailed paired t-test. n = 6. **p < 0.01, ***p < 0.001, ****p < 0.0001.

## Slide 4
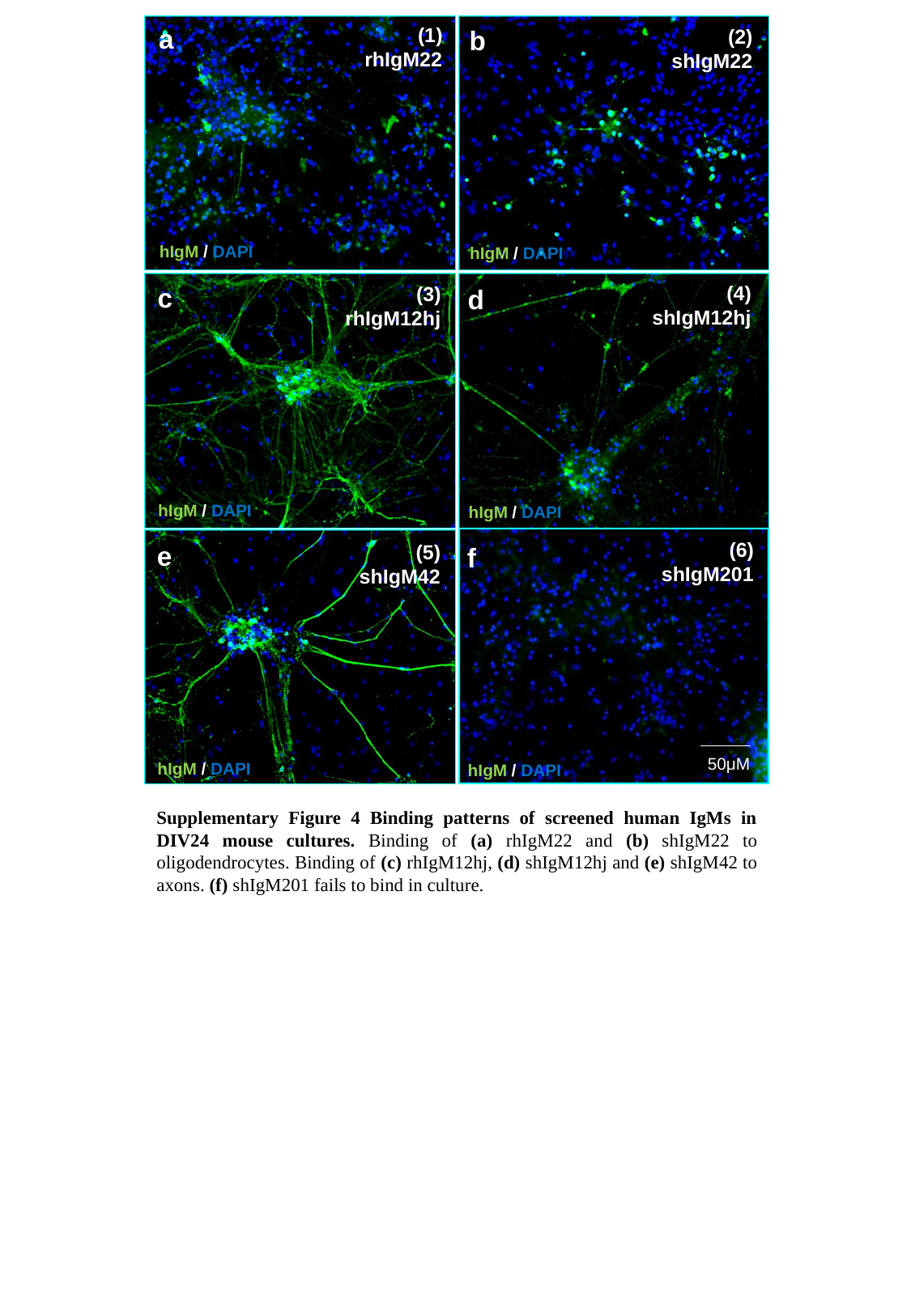

a
(1)
rhIgM22
b
(2)
shIgM22
hIgM / DAPI
hIgM / DAPI
(4)
shIgM12hj
c
(3)
rhIgM12hj
d
hIgM / DAPI
hIgM / DAPI
(6)
shIgM201
e
(5)
shIgM42
f
50μM
hIgM / DAPI
hIgM / DAPI
Supplementary Figure 4 Binding patterns of screened human IgMs in DIV24 mouse cultures. Binding of (a) rhIgM22 and (b) shIgM22 to oligodendrocytes. Binding of (c) rhIgM12hj, (d) shIgM12hj and (e) shIgM42 to axons. (f) shIgM201 fails to bind in culture.
